# The Italian multiple sclerosis register

**DOI:** 10.1007/s10072-018-3610-0

**Published:** 2018-11-13

**Authors:** Maria Trojano, Roberto Bergamaschi, Maria Pia Amato, Giancarlo Comi, Angelo Ghezzi, Vito Lepore, Maria Giovanna Marrosu, Paola Mosconi, Francesco Patti, Michela Ponzio, Paola Zaratin, Mario Alberto Battaglia, on behalf of the Italian Multiple Sclerosis Register Centers Group, D. Acquistapace, U. Aguglia, M.P. Amato, P. Annunziata, B. Ardito, C. Avolio, R. Balgera, F. Bandini, P. Banfi, P. Barone, P. Bellantonio, R. Bergamaschi, A. Bertolotto, P. Bertora, R. Bombardi, G. Bosco Zimatore, R.B. Bossio, P. Bramanti, V. Brescia Morra, A.M. Brioschi, M. Bruzzone, M. Buccafusca, V. Busillo, G. Caneve, L.M. Caniatti, L. Capone, F. Capone, A. Cappellani, D. Cargnelutti, G. Cavaletti, P. Cavalla, M.G. Celani, D. Centonze, L. Chiveri, R. Clerici, M. Clerico, E. Cocco, G. Comi, C. Comi, M.G. Coniglio, S. Cordera, F. Corea, A. Cortese, G. Costantino, S. Cottone, P. Crociani, F. D’Andrea, M.C. Danni, G. De Luca, D. de Pascalis, F. De Robertis, N. De Stefano, G. Di Battista, M. Di Napoli, M. Falcini, F. Fausto, M.T. Ferrò, C. Florio, M. Fortunato, C. Frittelli, S. Galgani, P. Gallo, M. Gatto, P. Gazzola, C. Geda, A. Giordano, F. Granella, M.G. Grasso, L.M.E. Grimaldi, D. Imperiale, L. Lo Russo, F.O. Logullo, A. Lugaresi, G. Lus, G. Maccarrone, D. Maimone, S. Malagù, R. Marconi, P. Maritato, L. Massacesi, M. Mazzoni, G. Meucci, M. Mirabella, S. Montepietra, D. Nasuelli, W. Neri, G. Orefice, S. Parodi, L. Pasquali, B. Passarella, F. Patti, M. Peresson, F. Perla, I. Pesci, C. Piantadosi, M.L. Piras, N.R. Pizio, C. Pozzilli, A. Protti, M. Pugliatti, R. Quatrale, M. Ragno, M. Ragno, M. Rezzonico, G. Ribizzi, M. Riva, M. Ronzoni, M.G. Rosso, M. Rottoli, M. Rovaris, G. Salemi, M. Salvetti, M. Santangelo, G. Santangelo, G. Santuccio, G. Santuccio, P. Sarchielli, E. Scarpini, G.P. Sechi, S. Severi, L. Sinisi, P. Sola, D. Spitaleri, T. Tassinari, G. Tedeschi, S. Tonietti, V. Torri Clerici, R. Totaro, S. Traccis, M. Trojano, M. Turla, A. Uccelli, M. Ulivelli, P. Valentino, M. Valeriani, S. Venturi, M. Vianello, M. Zaffaroni

**Affiliations:** 1 Department of Basic Medical Sciences, Neuroscience and Sense Organs, University of Bari “Aldo Moro” Policlinico, Italy Piazza Umberto I, Bari, Bari Italy; 2 Multiple Sclerosis Center, IRCCS Mondino Foundation, Pavia, Italy; 3 Department NEUROFARBA, MS Center AOU Careggi, University of Florence, Florence, Italy; 4 Neurology Department and INSPE-Institute of Experimental Neurology, Vita-Salute San Raffaele University, Milan, Italy; 5 Centro Studi Sclerosi Multipla, Ospedale di Gallarate, Gallarate, Va Italy; 6 Coreserach Center for Outcomes Research and Clinical Epidemiology, Pescara, Italy; 7 Istituto di Ricerche Farmacologiche Mario Negri IRCCS, Milan, Italy; 8 University of Cagliari Centro Sclerosi Multipla, Cagliari, Italy; 9 Department of Neurosciences G.F. Ingrassia, University of Catania, Catania, Italy; 10 Italian Multiple Sclerosis Foundation, Via Operai 40, Genoa, Italy; 11 Department of Life Sciences, University of Siena, Siena, Italy

**Keywords:** Multiple sclerosis, Register, Epidemiology, Quality of care

## Abstract

The past decade has seen extraordinary increase in worldwide availability of and access to several large multiple sclerosis (MS) databases and registries. MS registries represent powerful tools to provide meaningful information on the burden, natural history, and long-term safety and effectiveness of treatments. Moreover, patients, physicians, industry, and policy makers have an active interest in real-world observational studies based on register data, as they have the potential to answer the questions that are most relevant to daily treatment decision-making. In 2014, the Italian MS Foundation, in collaboration with the Italian MS clinical centers, promoted and funded the creation of the Italian MS Register, a project in continuity with the existing Italian MS Database Network set up from 2001. Main objective of the Italian MS Register is to create an organized multicenter structure to collect data of all MS patients for better defining the disease epidemiology, improving quality of care, and promoting research projects in high-priority areas. The aim of this article is to present the current framework and network of the Italian MS register, including the methodology used to improve the quality of data collection and to facilitate the exchange of data and the collaboration among national and international groups.

**Electronic supplementary material:**

The online version of this article (10.1007/s10072-018-3610-0) contains supplementary material, which is available to authorized users.

## Introduction

Disease registries are well recognized as powerful tools to provide meaningful information on the burden, natural history, and long-term safety and effectiveness of treatments in the “real-life” population of patients with chronic diseases [1, 2]. Registries can successfully operate by the creation of a network of reference centers and a good collaborative feeling either from clinicians or from patients to assure a high-quality data collection. Moreover, registries need important economical investment to build the structure of the database and the software, disseminate the tool among centers, and maintain and monitor its appropriate use. Registries are receiving increased attention for their potential role in policy-making or decision-making processes for the development of appropriate model of care [3]. In recent years, the worldwide availability of several large multiple sclerosis (MS) databases [4]—combined with a growing ability to collect, share, and analyze large amounts of data—are enabling the conduction of real-world observational studies aimed to identify MS prediction models for poor outcome and treatment response/failure, and to evaluate comparative and long-term effectiveness and safety of disease-modifying treatments in current use^2^, issues that cannot be addressed by RCTs. Data collected through registries should be used in prioritizing research and healthcare questions, to focus resources on these high-priority areas would likely accelerate progress in MS and to better leverage limited resources.

MS clinical data sharing initiative has a longstanding tradition in Italy. In 2000, the Italian collection of MS clinical data started at different Italian MS centers in the framework of the Italian Multiple Sclerosis Database Network (MSDN) [5, 6]. This network used the iMed© software’s system, a clinical database where more than 500 variables were collected [7].

Within this frame and in line with the International MS research strategic agenda, since 2013, the Italian MS Society representing people with MS (Associazione Italiana Sclerosi Multipla—AISM) together with its foundation (Fondazione Italiana Sclerosi Multipla—FISM) have been engaged in promoting and funding data sharing initiatives. In 2014, FISM, in collaboration with the Italian MS clinical centers, promoted and funded the creation of the Italian MS Register, a project in continuity with the existing MSDN-iMed© software’s clinical database collection.

One of the first expressed purposes of Italian MS Register is to create an organized multicenter structure to collect data of all MS patients (a near population-level) followed in the larger number of Italian MS centers.

The Italian MS Register aims to address high-priority areas pertaining to

- public healthcare area: quality of care, health optimization such as economical optimization, social and welfare information, access to healthcare treatments and healthcare services
- research area: epidemiology, rare MS disease forms such as, primary progressive (PP) MS, pediatric MS as well as early and preclinical/subclinical disease stages represented by clinically isolated syndromes (CIS) and radiologically isolated syndromes (RIS), treatment optimization such as prognostic factors and predictive models of disease course, adherence to treatments, treatment efficacy, and safety.

The aim of this article is to present the current framework and network of the Italian MS register, including the method of work used to improve the quality of data collection and to facilitate the exchange of data and the collaboration among national and international groups.

## Framework and network of the Italian MS register

After completing preparatory phase, the Italian MS Register officially started in January 2015. It is fully financed by FISM and AISM, the MS national charity in Italy.

### Governance

The governance of the Italian MS Register includes an Executive Committee (chaired by AISM and University of Bari) with the administrative and organizational role and a Scientific Committee which oversees the scientific initiatives, promotes specific strategic projects, and approves requests of access to centralized data for further research projects. Scientific Committee includes clinicians, methodologists, representatives of MS centers, and of the Italian Neurological Society (SIN).

Technical and Administrative Infrastructure (TAI) is coordinated by FISM accordingly with a Technical Methodological Structure (TMS) based at IRCCS Istituto Ricerche Farmacologiche Mario Negri-Coresearch.

In order to increase the quality of the data collected, a group of 12 research assistants has been ad hoc trained for the project with the objective to foster the collection of good quality data in the Italian MS centers. Each assistant was allocated to one or more centers (depending of the size of the MS center). Research assistants monthly report the activity to the TMS, and at least three/times year, they are involved in meeting to discuss data collected. To meet the strategic priorities of the Italian MS Register, relevant stakeholders, including industries, are engaged through advisory forum.

Through the website of the AISM, each participating center can propose research projects addressing one of the high-priority areas of the register. All the projects are discussed by Scientific Committee before their implementation.

### Ethics committee

The Italian MS register was approved by the Ethics Committee of the University of Bari (Italy) as coordinator center and the local ethics committee of all participant centers. Each individual with a diagnosis of MS enrolled is required to sign a written informed consensus to enter into the register.

Since in some of the participant centers data were collected before the starting of the Italian MS Register (through iMed© or other data-entry), according to the local laws and regulations, data collected retrospectively can be also included without informed consent.

### MS centers

The medical assistance to MS patients in Italy is mainly delivered by 236 qualified MS centers. The ambition of the Italian Register is to completely represent the MS reality in Italy, so all the 236 Italian MS centers have been contacted by AISM/FISM in order to explore their willingness to participate to the Register project. Of these, 141 (60%) declared the willingness to participate, 47 of them were already using the iMed© and were asked to reverse their data in the new Register. The remaining MS centers were warmly encouraged to join in the Register. The centers were required to include all the MS cases in the Register, to transfer a standardized set of data using local or central database, and to inform people with MS about the Register. Participation in Italian MS Register is voluntary both from the neurologist’s and the patient’s side.

### Data collection

The Scientific Committee agreed, by consensus, on a compulsory common minimum dataset (MDS) consisting of selected information according to the principles of relevance to ensure the collection of sufficient data for the clinical characterization of the single patient. The list of the mandatory variables of interest, identified on the basis of the existing guidelines and the recommendations of the Scientific Committee, ensures

- participation of a large and representative number of centers
- easy and simple data collection
- ability to each center to achieve maximum completeness and quality of data
- possible development of linkage procedures with regional information flows of health administrative data (hospital discharges, prescription drugs, ticket exemptions, register of patients, outpatient specialist)

This MDS may be completed with an extension to optional information already available in the iMed© computerized medical folder [8, 9] (Table 1).

**Table 1:** Simplified description of the information collected by the Italian MS Register with indications of the mandatory variables included in the MDS (in bold) and the optional variables (in italic). A detailed description of the MDS variables is reported in Appendix 1

Section	Sub-Section	Description
**Baseline**	**Clinical center**	**Identification**
	**Patient**	**Identification and encryption of personal data** **State of Life** **Record creation date**
	**Onset MS**	**Date, symptoms, course (including RIS)**
	**Diagnosis**	**Date, Mc Donald Criteria 2010 and 2017** [8, 9]
**Follow-up (FU)**	**Visits**	**Date, EDSS, course**
	**Relapses**	**Date, duration, functional systems involved, severity, recovery, steroid treatment**
	**Treatments**	**Start date, end date, dosage, administration routes, disease modifying or symptomatic therapies, discontinuation cause (if applicable)**
	**Adverse events**	**Reporting severe adverse events and adverse events (using MedDRA coding system)**
*Paraclinical tests*	*Magnetic Resonance Imaging*	*Date, CNS region, presence and number of T2, T1 and, T1 Gd + lesions, McDonald’s Criteria (2017) for space and time dissemination*
	*Cerebrospinal fluid*	*Date, routine, oligoclonal bands (presence/absence)*
	*Evoked potentials*	*Date, visual evoked potentials(VEP), upper and lower somatosensory and motor evoked potentials (SEP and MEP), brainstem auditory evoked potentials (BAEP)*
	*Laboratory tests*	*Date, routine, hematologic, virological, immunological, thyroid function*
*Clinical events*	*History*	*Family history, pregnancies, comorbidities*

During its first years, the Italian MS Register was based on a client–server solution, thus requiring hardware, software installed on each computer (iMed© software’s), and local IT support. At the end of 2016, it has been decided that the Italian MS Register should go web-based to also facilitate the interface with other national and international databases. This improvement of data collection was implemented from 2017. A data collection website is now available at: https://registroitalianosm.it/ where each center can enter the data through a personalized password. Only cases with MDS properly completed are accepted in the database. It is noteworthy that in the Italian MS register, it is possible to check the presence of a unique valid code identifier, through the patient encrypted fiscal code, in order to overcome one of the main issues of large population registers that is the inclusion of the same patient by two or more MS centers where the patient himself had turned to their own care pathway.

### Data monitoring

Data are centrally monitored in order to guarantee a high quality of information collected. Centers are periodically contacted with ad hoc reports with queries on the missing data or inconsistencies among the variables collected.

Several quality control tools have been implemented in order to increase the quality and generalizability of data collected. Every 2/3 months per year, all the centers are reached with a report regarding all the data collected and a tailored report regarding each center. Quality controls regard

- dates: presence/absence, completeness, anomalies and consistency among all the data collected in the dataset
- completeness: overall evaluation of the completeness level of the variables included
- accuracy: proportion of variables with value corresponding to their range
- consistency: congruency with other variables

Moreover, a set of seven performance indicators has been identified and adopted with the aim to improve the quality, completeness of the survey, timeliness, generalization, and representativeness of the collected data (Table 2). For each examined indicator or domain each participating center was awarded with a score of 5 for the highest performance, while lower scores of 4 to 1 were attributed for progressively lower performance.

**Table 2:** Performance indicators

Score	Optimal reference requirement	Requirement calculation mode	Assessment of the data quality score (quality metrics)
Update Center adherence to periodic central database update	Participating centers are required to upload data to the central server every 6 months	Interval between the update report date and the last upload date	Within 6 months 5 points > 6 months and ≤ 1 year 4 points > 1 year and ≤ 2 years 3 points > 2 years and ≤ 3 years 2 points Over 3 years: 1 point
N. Patients-year Sample size by center	Number of patients-year in the top quintile	The number of patients per year is calculated as the sum of the follow-up years* of each patient *Interval in years between last and first visit date recorded	Attribution based on quintile distribution: Within V quintile 5 points Within IV quintile 4 points Within III quintile 3 points Within II quintile 2 points Within I quintile 1 point
Patients with FUP ≥ 5 years Sample size by center with prospective clinical follow-up ≥ 5 years	% of patients with follow-up ≥ 5 years per center > 80%.	% of patients with follow-up ≥ 5 years per center	> 80% and ≤ 100% 5 points > 60% and ≤ 80% 4 points > 40% and ≤ 60% 3 points > 20% and ≤ 40% 2 points > 0% and ≤ 20% 1 point
Active patients Patients in active status, i.e. at least one visit and/or contact with the center in the last two years	% of patients in active status per center > 80%	% of patients in active status per center	> 80% and ≤ 100% 5 points > 60% and ≤ 80% 4 points > 40% and ≤ 60% 3 points > 20% and ≤ 40% 2 points > 0% and ≤ 20% 1 point
VISIT every 6 months Semi-annual visit rates	At least one visit every 6 months in the follow-up period in >80% of patients in each center	% of patients with at least one visit every 6 months in the follow-up period for center	> 80% and ≤ 100% 5 points > 60% and ≤ 80% 4 points > 40% and ≤ 60% 3 points > 20% and ≤ 40% 2 points > 0% and ≤ 20% 1 point
EDSS every 6 months Semi-annual EDSS assessment rates	At least one EDSS assessment every 6 months in the follow-up period in > 80% of patients in each center	% of patients with at least one EDSS assessment every 6 months in the follow-up period for center	> 80% and ≤ 100% 5 points > 60% and ≤ 80% 4 points > 40% and ≤ 60% 3 points > 20% and ≤ 40% 2 points > 0% and ≤ 20% 1 point
I° visit within I°year from onset First visit within 1 year of the disease onset	At least one visit within one year of the disease onset in >80% of patients in each center	% of patients with at least one visit within 1 year of the disease onset for center	> 80% and ≤ 100% 5 points > 60% and ≤ 80% 4 points > 40% and ≤ 60% 3 points > 20% and ≤ 40% 2 points > 0% and ≤ 20% 1 point

## State-of-the art of the Italian MS register

### MS centers

**Fig. 1:**
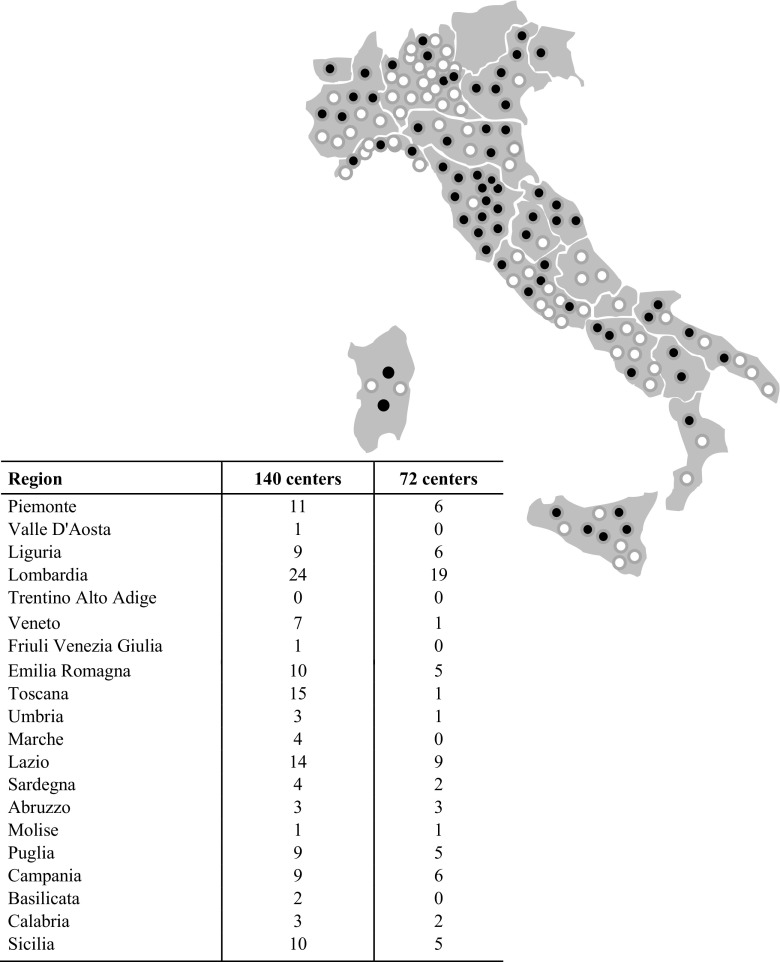
Distribution of the 140 centers participants (black circle) to Italian MS Register and of 72 centers (white circle) with actual data transfer to the central database

As reported in Fig. 1, 140 out of 236 contacted centers (60%) declared the willingness to participate (last update May 2018), and 103 completed their ethics committee process for approval and are ready to participate to the data collection. On May 2018, 72 MS centers effectively contributed uploading data. The geographic distribution of the centers is reported in Fig. 1.

### Sample

**Fig. 2:**
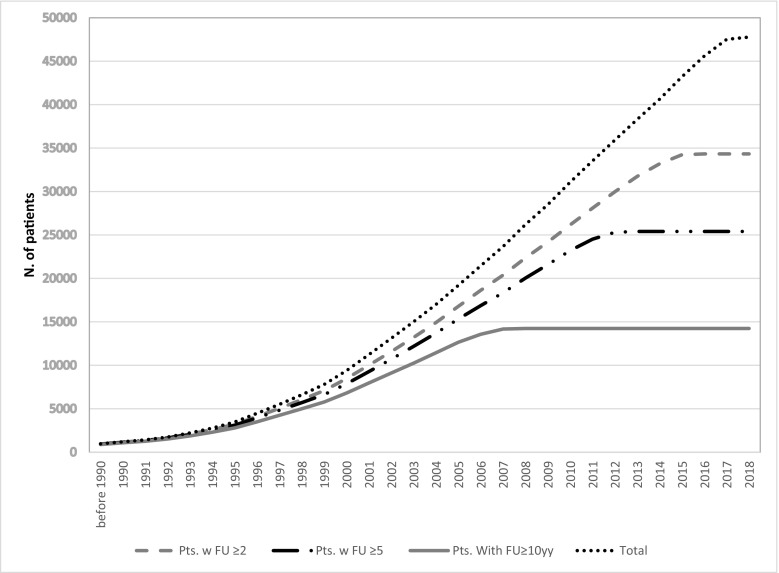
Cumulative recruitment of patients per year of entry into the cohort in relation to follow-up (FU) duration (in years) (Appendix 2 data in detail)

The increasing temporal trends of the total cohort and sub-cohorts with different follow-up duration (≥ 2.0, 5.0, and 10 years) by May 2018 are reported in Fig. 2. The same patient was registered in two or more sites in 6.1% of cases.

The data counts of the mandatory variables of 44,636 people with MS included in the Register by May 2018 are summarized in Table 3.

**Table 3:** Characteristics of 44,636 MS people with MS enrolled in the Italian register

	n = (%)	Mean ± SD in years
Female	32,296 (67)	
Age at onset (years)		30.5 ± 10.5
Delay between diagnosis and MS onset (years)		3.5 ± 5.8
Disease course at the last visit		
Missing Clinically Isolated syndrome Relapsing-remitting Secondary progressive Primary progressive w or w/o relapses	828 (1.7) 2711 (5.6) 35,619 (74.0) 6115 (12.7) 2879 (6.0)	
Number of visits per patient		13.4 ± 14.8
Number of registered DMD prescriptions	107,539	
Number of registered relapses	160,419	
Number of registered EDSS	570,640	
Number of registered EDSS per patient		11.8 ± 13.6
Number of registered MRI	314,994	
Number of registered MRI per patient		6.5 ± 7.1

### Consistency, completeness, and quality control of data

Twenty-eight variables included in the MDS were selected to evaluate the level of completeness. The percentage of completeness of the examined variables ranged between 30% (duration in days of relapse) and 100% (for 10 of the 28 variables) (Appendix 3).

An example of the quality control for the accuracy and consistency of event dates (presence/absence and/or anomalies among dates) is reported in Appendix 4. The range of the accuracy and consistency was between 96% (Date First Visit at the center) and 100% (for 9 of 15 variables).

**Fig. 3:**
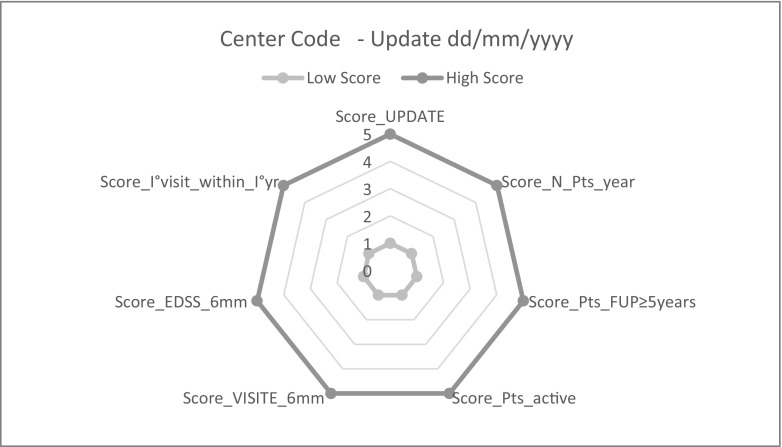
Quality of data collected. Legend (see also Table 2 for more details). Score_UPDATE means adherence to periodic central database update; Score_N._Pts_year means sample size by center; Score:_Pts_with FUP ≥ 5 years means sample size by center with prospective clinical follow-up ≥5 years; Score:_active Pts means patients in active status, i.e., at least one visit and/or contact with the center in the last 2 years; Score:_VISIT every 6 months means semi-annual visit rates; Score:_EDSS every 6 months means semi-annual EDSS assessment rates; Score:_I°visit_within_I° yr from onset means first visit within 2 year of the disease onset.

Finally, the graphic representation of seven performance indicators is reported in Fig. 3. Every 6 months, each participating center receives a report where data and performance indicators of its own center are benchmarked with the whole sample: in this way, each center can assess the most critical performances and the level of improvement with time.

### Research activity

A standardized process for applications of research project has been developed. The applications are submitted through https://www.aism.it/bandiregistro website after registration of the project manager. Firstly, the feasibility of proposals (i.e., variables availability, completeness of data, size of the sample, etc.) is assessed by TMS. Then, the members of the Scientific Committee assess the proposals according to scientific quality, value of the project, and alignment with priority areas of the Register. Up to now, 14 research projects addressing one of the high-priority areas of the Register have been proposed and discussed by the Scientific Committee. The 12 approved projects cover the following research areas: epidemiology; prognostic factors, and predictive models of disease course: adherence, efficacy, and safety of the treatments.

## Discussion

Patient registries gather valuable long-term patients’ information from the real world which are useful to a wide range of purposes: to better define the disease epidemiology within specific geographic areas, understand the social and economic impact of a disease; to provide the national regulatory authorities findings to make relevant decisions about focused healthcare programs improving quality of healthcare; to improve the care of MS patients; to provide updated information on the evolution of MS in large cohorts of patients; and to better evaluate the impact of DMTs in real life.

Concerning MS, some large-scale national and international registries and databases are currently in use with different aims and designs [4, 10–17].

Italy is an area with medium-high prevalence of MS, which is estimated from 122 to 232 cases/100,000 in the mainland and Sicily and from 280 to 317 cases/100,000 in Sardinia. Applying an extrapolation to the Italian population in 2015, about 110,000 MS patients are estimated in Italy [18].

In 2000, the Italian collection of MS clinical data started at different Italian MS centers in the framework of MSDN [5, 6] allowing the production of a number of scientific research papers [19–25]. In 2014 FISM, in collaboration with the Italian MS clinical centers, promoted the creation of the first Italian MS Register.

In light of a practical guidance in setting up patient registries [1], the Italian Register answers four main expectancies: (1) it allows to collect reliable data for monitoring and evaluating patient care, and to promote research projects (the Why—mission and goals); (2) it has a clear and functional governance structure that aligns the objectives of the register and agrees with stakeholders (the Who—stakeholders and funding); (3) it can easily collect specific data by means of a minimum data set which reliability and validity has been carefully verified, thanks to a sound network of Italian MS centers (the What—type and content); (4) patients are well identified and recruited by centers with proven expertise in the field of MS, whose data are protected, handled, and analyzed by a technical structure with proven expertise in the field data management (the How—identification and recruitment of patients, data handling).

The mission of the Italian Register project is not limited to assure quality healthcare for patients with MS but also to promote research projects addressing high-priority issues. At present, 12 research projects are ongoing using the data collected through the register.

With reference to a recent workshop on MS patient registries, we think that the so conceived Italian Register is already responding to the main recommendations expressed by the experts [25]. We detail below the seven recommendations, how we currently deal with them, and what we aim to do to improve their managing and development.

Recommendation 1 (Create a federated network of cohorts). Our Italian MS Register is based on an already existing network of MS centers. Anyway, 140 out of the 236 Italian MS centers agreed to join in the Register project; thus, at the present, the Italian Register contains the records of about 50,000 MS patients, which is 40% of the expected coverage of Italian MS population. MS centers that did not contribute to the initial phase of data collection and revision are now invited to take part into the initiative to ensure the full coverage of the Italian MS population.

Recommendation 2 (Standardize data collection and management). We have defined a set of MDS required to include for each patient to ensure the collection of sufficient data for their characterization. MDS allows us to record data in a standardized way. The support of research assistants, trained ad hoc*,* improves the management of data and their quality*.* In the future, the activity of research assistants should be strengthened and extended to the majority of MS centers, in order to enrich the database not only quantitatively but also qualitatively.

Recommendation 3 (Identify and prioritize research questions). Our Scientific Committee has identified research questions, giving them different priorities. The topics on which researches will be focused might change in future, according to the debate with MS centers, scientific societies, health authorities, and stakeholders.

Recommendation 4 (encourage collection of physician- and patient-reported outcomes). We will encourage, through scientific societies and media tools, people involved in MS management to join in the Italian Register project. Neurologists could be further encouraged by the prospective to make use of the register network to exploit researches addressing one of the high-priority areas of the Register.

Recommendation 5 (encourage technological innovation). Our Register already employs an ad hoc database. In a short time, specific additional database (for pediatric MS, pregnancy, MRI) will be available, as well as a dedicated Internet platform to facilitate data inclusion and transmission.

Recommendation 6 (develop a universal informed consent process). We have defined an informed consensus that must be signed to enter in the Italian Register. Anyway, given the wide differences among the laws that rule ethical issues in different nations, to get to informed consent universally shared is quite unrealistic.

Recommendation 7 (provide sustainable funding). The Register needs important economical investments to be disseminated and maintained. At the moment, the Register is funded by FISM and other donors, including pharmaceutical companies engaged through advisory forum.

For several research purposes, although cohort studies and registries typically include considerable numbers of patients, analyses are often limited by poor statistical power owing to insufficient numbers of patients. This is true for many aspects of real-world analysis.

As a consequence, in the past 10 years, a fruitful collaboration between MSBase and some Italian centers was established: individual MS centers (10/140), belonging to the MS Database Network, and currently belonging to the Italian MS Registry, shared, on annual or biannual basis, their data with the MSBase Registry for collaborative scientific projects. Currently, since an Italian MS Registry has been set up with new rules, the collaboration with MSBase, and other European registries will occur through data sharing for specific and agreed projects. Individual centers will remain free to collaborate with MSBase or other registries for specific projects, but not by releasing data regularly and independently of them, and they must notify their participation in these projects to the Scientific Committee in order to avoid overlap with projects already underway in the Italian Registry. This new approach will extend collaborations, keeping the identity of the Italian registry separate from that of MSBase or other registries.

Over the past 3 years, representatives of five leading MS registries (including the Italian, Danish and Swedish MS registries, French Observatoire Française de Sclérose en Plaque network and the international MSBase) have been working together to explore opportunities for data sharing, so called BigMS Data group project. Combined, the five registries collect longitudinal data on > 150,000 patients with MS. To date, the BigMS Data group has identified and agreed on a minimal set of parameters and initiated three pilot projects with joint data.

These efforts need to overcome challenges of technical, ethical, legal, and political nature, but over long term, they are hoped to be of significance. The key findings in international registries should be also utilized in conjunction with data from clinical trials to optimize treatment and improve long-term outcomes.

In the next future, it would be desirable a larger use of MS disease registries for the post-marketing drug safety assessment (i.e., post-approval Safety—PASS- project recently proposed by EMA). Indeed, large disease registries, unlike drug registries, can include information not only on products or procedures of interest but also on similar patients who receive other treatments, other procedures, or no treatment for the same clinical indications allowing a better evaluation of event rates, consequences of long-term use, and/or effects of various combinations and sequencing of treatments. Moreover, the use of disease registries may provide a better understanding of the effects of comorbidity on effectiveness and safety of DMTs.

## Electronic supplementary material

ESM 1 (DOCX 52 kb)

## References

[1] Gliklich RE, Leavy MB (2014). Registries for evaluating patient outcomes: a User's Guide.

[2] Trojano M, Tintore M, Montalban X, Hillert J, Kalincik T, Iaffaldano P, Spelman T, Sormani MP, Butzkueven H (2017). Treatment decisions in multiple sclerosis—insights from real-world observational studies. Nat Rev Neurol.

[3] De Groot S, van der Linden N, Franken MG (2017). Balancing the optimal and the feasible: a practical guide for setting up patient registries for the collection of real-world data for health care decision making based on Dutch experiences. Value Health.

[4] Flachenecker P, Buckow K, Pugliatti M, Kes VB, Battaglia MA, Boyko A, Confavreux C, Ellenberger D, Eskic D, Ford D, Friede T, Fuge J, Glaser A, Hillert J, Holloway E, Ioannidou E, Kappos L, Kasilingam E, Koch-Henriksen N, Kuhle J, Lepore V, Middleton R, Myhr KM, Orologas A, Otero S, Pitschnau-Michel D, Rienhoff O, Sastre-Garriga J, Schyns-Liharska T, Sutovic D, Thalheim C, Trojano M, Vlasov YV, Yaldizli Ö, for the EUReMS Consortium* (2014). Consortium EU. Multiple sclerosis registries in Europe—results of a systematic survey. Mult Scler.

[5] Trojano M, Granieri E, Rosati G et al (2003) In: abstracts from the 19th congress of the European Committee for Treatment and Research in Multiple Sclerosis, Milan, Italy 14640129

[6] Trojano M, Liguori M, Paolicelli D, Zimatore GB, de Robertis F, Avolio C, Giuliani F, Fuiani A, Livrea P, Southern Italy MS Group (2003). An independent post-marketing study in southern Italy. Mult Scler.

[7] Mechati S, Peyro-St-Paul H (2001). iMed: a new electronic database for monitoring patients with multiple sclerosis. Mult Scler.

[8] Polman CH, Reingold SC, Banwell B, Clanet M, Cohen JA, Filippi M, Fujihara K, Havrdova E, Hutchinson M, Kappos L, Lublin FD, Montalban X, O'Connor P, Sandberg-Wollheim M, Thompson AJ, Waubant E, Weinshenker B, Wolinsky JS (2011). Diagnostic criteria for multiple sclerosis: 2010 revisions to the McDonald criteria. Ann Neurol.

[9] Thompson AJ, Banwell BL, Barkhof F, Carroll WM, Coetzee T, Comi G, Correale J, Fazekas F, Filippi M, Freedman MS, Fujihara K, Galetta SL, Hartung HP, Kappos L, Lublin FD, Marrie RA, Miller AE, Miller DH, Montalban X, Mowry EM, Sorensen PS, Tintoré M, Traboulsee AL, Trojano M, Uitdehaag BMJ, Vukusic S, Waubant E, Weinshenker BG, Reingold SC, Cohen JA (2018). Diagnosis of multiple sclerosis: 2017 revisions of the McDonald criteria. Lancet Neurol.

[10] Koch-Henriksen N, Magyari M, Laursen B (2015). Registers of multiple sclerosis in Denmark. Acta Neurol Scand.

[11] Myhr KM, Grytten N, Torkildsen Ø, Wergeland S, Bø L, Aarseth JH (2015). The Norwegian multiple sclerosis registry and biobank. Acta Neurol Scand.

[12] Hillert J, Stawiarz L (2015). The Swedish MS registry—clinical support tool and scientific resource. Acta Neurol Scand.

[13] Marrie RA, Cutter G, Tyry T, Campagnolo D, Vollmer T (2007). Validation of the NARCOMS registry: diagnosis. Mult Scler.

[14] Confavreux C, Compston DA, Hommes OR, McDonald WI, Thompson AJ (1992). EDMUS, a European database for multiple sclerosis. J Neurol Neurosurg Psychiatry.

[15] Cotton F, Kremer S, Hannoun S, Vukusic S, Dousset V, Imaging Working Group of the Observatoire Français de la Sclérose en Plaques (2015). OFSEP, a nationwide cohort of people with multiple sclerosis: consensus minimal MRI protocol. J Neuroradiol.

[16] Stuke K, Flachenecker P, Zettl U (2008). MS register in Germany: update 2007. J Neurol.

[17] Butzkueven H, Chapman J, Cristiano E, Grand’Maison F, Hoffmann M, Izquierdo G, Jolley D, Kappos L, Leist T, Pöhlau D, Rivera V, Trojano M, Verheul F, Malkowski JP (2006). MSBase: an international, online registry and platform for collaborative outcomes research in multiple sclerosis. Mult Scler.

[18] Battaglia MA, Bezzini D (2017). Estimated prevalence of multiple sclerosis in Italy in 2015. Neurol Sci.

[19] Trojano M, Russo P, Fuiani A, Paolicelli D, di Monte E, Granieri E, Rosati G, Savettieri G, Comi G, Livrea P, MSDN Study Group (2006). The Italian multiple sclerosis database network (MSDN): the risk of worsening according to IFNbeta exposure in multiple sclerosis. Mult Scler.

[20] Trojano M, Pellegrini F, Fuiani A, Paolicelli D, Zipoli V, Zimatore GB, di Monte E, Portaccio E, Lepore V, Livrea P, Amato MP (2007). New natural history of interferon-beta-treated relapsing multiple sclerosis. Ann Neurol.

[21] Bergamaschi R, Quaglini S, Trojano M (2007). Early prediction of the long-term evolution of multiple sclerosis: the Bayesian risk estimate for multiple sclerosis (BREMS) score. J Neurol Neurosurg Psychiatry.

[22] Trojano M, Pellegrini F, Paolicelli D, Fuiani A, Zimatore GB, Tortorella C, Simone IL, Patti F, Ghezzi A, Zipoli V, Rossi P, Pozzilli C, Salemi G, Lugaresi A, Bergamaschi R, Millefiorini E, Clerico M, Lus G, Vianello M, Avolio C, Cavalla P, Lepore V, Livrea P, Comi G, Amato MP, Italian Multiple Sclerosis Database Network (MSDN) Group (2009). Real-life impact of early interferon beta therapy in relapsing multiple sclerosis. Ann Neurol.

[23] Trojano M, Pellegrini F, Paolicelli D, Fuiani A, Zimatore GB, Tortorella C, Simone IL, Patti F, Ghezzi A, Portaccio E, Rossi P, Pozzilli C, Salemi G, Lugaresi A, Bergamaschi R, Millefiorini E, Clerico M, Lus G, Vianello M, Avolio C, Cavalla P, Iaffaldano P, Direnzo V, D'Onghia M, Lepore V, Livrea P, Comi G, Amato MP, Italian Multiple Sclerosis Database Network Group (2009). Italian multiple sclerosis database network group. Post-marketing of disease modifying drugs in multiple sclerosis: an exploratory analysis of gender effect in interferon beta treatment. J Neurol Sci.

[24] Bergamaschi R, Quaglini S, Tavazzi E, Amato MP, Paolicelli D, Zipoli V, Romani A, Tortorella C, Portaccio E, D’Onghia M, Garberi F, Bargiggia V, Trojano M (2016). Immunomodulatory therapies delay disease progression in multiple sclerosis. Mult Scler.

[25] Bebo BF, Fox RJ, Lee K (2017). Landscape of MS patient cohorts and registries: recommendations for maximizing impact. Mult Scler.

