# Supplementary material for: The Italian multiple sclerosis register
Source: Neurol Sci. 2018 Nov 13;40(1):155–65. doi: 10.1007/s10072-018-3610-0 (PMC6329744; doi:10.1007/s10072-018-3610-0)
Supplement: Supplementary file 1 — (DOCX 52 kb) [file 10072_2018_3610_MOESM1_ESM.docx]

**Appendix 1** List of the mandatory MDS variables by baseline and follow-up data

| **BASELINE - CENTER** | **DESCRIPTION** |
| --- | --- |
| Center code | Center identification code attributed by the Technical Methodological Structure |
| Center name – Short | Short name of the center attributed by the Technical Methodological Structure |
| Full name of the center | Institutional denomination of the center |
| **BASELINE - PATIENT** |  |
| Patient code | Unique patient identification code automatically assigned by the computerized system of the center |
| Health Insurance code | Encrypted fiscal code of the patient |
| Date of creation record | Date of patient inclusion in the information system of the center - dd/mm/yyyy |
| Date of birth | Date of birth of the patient - dd/mm/yyyy |
| Sex | F=female; M=male |
| Birth city | Birth city of the patient |
| Deceased | 1="Yes"; 0 or Missing="No" |
| Date of death | If deceased = 1 indicate date of death - dd/mm/yyyy |
| Death related to MS | If death related to MS = 1; 0 or Missing = death not related to SM |
| Country of birth | Country birth code |
| City | City of residence |
| Region | Region of residence |
| Country | Country code of residence |
| **BASELINE - ONSET AND DIAGNOSIS** |  |
| Patient Code | Unique patient identification code automatically assigned by the computerized system of the center |
| Date of progression | Date of beginning of progression - dd/mm/yyyy |
| Date of diagnosis | Valid diagnosis date - dd/mm/yyyy |
| Date of onset | Valid date of onset - dd/mm/yyyy |
| Date of first visit at the center | Valid date - dd/mm/yyyy |
| Brainstem-Cerebellum symptoms at onset | 1="Yes"; 0 or Missing="No" |
| Optic pathways symptoms at the onset | 1="Yes"; 0 or Missing="No" |
| Spinal cord symptoms at the onset | 1="Yes"; 0 or Missing="No" |
| Supratentorial symptoms at the onset | 1="Yes"; 0 or Missing="No" |
| Other symptoms at the onset | Text, specific |
| Progression from onset | 1="Yes"; 0 or Missing="No" |
| Confirmed diagnosis according to McDonald 2010 | 1="Diagnosis di MS"; 2="Possible MS"; 3="No MS"; 0="Missing" |
| First MS Course | First MS course (SP MS; PP MS; PR MS; RR MS; Benign; Malignant; CIS) |
| Second MS Course | Second MS course (SP MS; PP MS; PR MS; RR MS; Benign; Malignant; CIS) |
| Third MS Course | Third MS course (SP MS; PP MS; PR MS; RR MS; Benign; Malignant; CIS) |
| Date of first MS course | Date first MS course - dd/mm/yyyy |
| Date of second MS course | Date second MS course - dd/mm/yyyy |
| Date of third MS course | Date third MS course - dd/mm/yyyy |
| **FOLLOW-UP VISITS** |  |
| Patient code | Unique patient identification code automatically assigned by the computerized system of the center |
| Visit code | Patient visit identification number |
| MS course | MS course (SP MS; PP MS; PR MS; RR MS; Benign; Malignant; CIS) |
| Date of visit | Visit date - dd/mm/yyyy |
| EDSS | Score EDSS |
| **FOLLOW-UP RELAPSES** |  |
| Patient code | Unique patient identification code automatically assigned by the computerized system of the center |
| Duration in days relapse | Number |
| Date of relapse | Start date relapse - dd/mm/yyyy |
| Affected functional system Bowel bladder | 1="Yes"; 0 or Missing="No" |
| Affected functional system Brainstem | 1="Yes"; 0 or Missing="No" |
| Affected functional system Cerebellum | 1="Yes"; 0 or Missing="No" |
| Affected functional system Psychic/mental functions | 1="Yes"; 0 or Missing="No" |
| Affected functional system Other | 1="Yes"; 0 or Missing="No" |
| Affected functional system Pyramidal tract | 1="Yes"; 0 or Missing="No" |
| Affected functional system Sensitive | 1="Yes"; 0 or Missing="No" |
| Affected functional system Visual functions | 1="Yes"; 0 or Missing="No" |
| Recovery after relapse | 1="Complete"; 2="Partial"; 3="None"; 0="Unknow" |
| Cortisonic treatment performed | 1="Yes"; 2="No"; 0 or Missing="Unknown" |
| Outpatient treatment | 1="Yes"; 0 or Missing="No" |
| Hospital treatment | 1="Yes"; 0 or Missing="No" |
| Impact ADL Functions | 1="Yes"; 2="No"; 0 or Missing="Unknown" |
| Severity | 1="Mild"; 2="Moderate"; 3="Severe"; 0 or Missing="Unknown" |
| **FOLLOW-UP DRUGS** |  |
| Patient code | Unique patient identification code automatically assigned by the computerized system of the center |
| Prescription code | Therapeutic prescription identification number |
| Name of the drug | Valid name of the drug - coded list |
| MS treatment specific | 1="Yes - Specific"; 0 ="No - Symptomatic" |
| Reason for discontinuation | 1= Adverse Event; 2= Allergic reaction; 3= Lack of efficacy; 4= Lack of tolerance, 5= Non adherence; 6= Patient Choice/Convenience; 7= Persistence of Relapse; 8= Persisting MRI activity; 9= Pregnancy Confirmed; 10= Pregnancy Planned; 11= Progression of disease; 12= Progression of EDSS; 13= Scheduled Stop |
| Date of end of treatment | Treatment end date - dd/mm/yyyy |
| Date of start of treatment | Treatment start date - dd/mm/yyyy |

**Appendix 2** Cumulative recruitment of patients per year of entry into the cohort, details of data reported in Figure 3 (N. 339 missing value)

| **Year of entry into the cohort** | **Patients with FU ≥2** | **Patients with FU ≥5** | **Patients with FU ≥10yy** | **Total** |
| --- | --- | --- | --- | --- |
| Before 1990 | 936 | 926 | 885 | 973 |
| 1990 | 1157 | 1143 | 1080 | 1197 |
| 1991 | 1366 | 1345 | 1258 | 1419 |
| 1992 | 1666 | 1634 | 1520 | 1742 |
| 1993 | 2096 | 2046 | 1881 | 2216 |
| 1994 | 2593 | 2517 | 2299 | 2771 |
| 1995 | 3225 | 3118 | 2793 | 3485 |
| 1996 | 4107 | 3961 | 3506 | 4476 |
| 1997 | 5052 | 4832 | 4252 | 5516 |
| 1998 | 6048 | 5714 | 4996 | 6613 |
| 1999 | 7093 | 6636 | 5778 | 7798 |
| 2000 | 8482 | 7879 | 6818 | 9395 |
| 2001 | 10018 | 9284 | 7974 | 11243 |
| 2002 | 11585 | 10710 | 9113 | 13112 |
| 2003 | 13216 | 12185 | 10246 | 15020 |
| 2004 | 14929 | 13712 | 11451 | 17004 |
| 2005 | 16790 | 15332 | 12655 | 19202 |
| 2006 | 18632 | 16875 | 13572 | 21455 |
| 2007 | 20386 | 18353 | 14171 | 23678 |
| 2008 | 22366 | 20035 | 14238 | 26202 |
| 2009 | 24182 | 21593 | 14238 | 28497 |
| 2010 | 26160 | 23208 | 14238 | 31078 |
| 2011 | 28086 | 24513 | 14239 | 33528 |
| 2012 | 29970 | 25305 | 14239 | 35924 |
| 2013 | 31748 | 25402 | 14239 | 38306 |
| 2014 | 33192 | 25402 | 14239 | 40631 |
| 2015 | 34235 | 25402 | 14239 | 43207 |
| 2016 | 34326 | 25402 | 14239 | 45594 |
| 2017 | 34326 | 25402 | 14239 | 47513 |
| 2018 | 34326 | 25402 | 14239 | 47771 |

**Appendix 3** Percentage of completeness of the MDS

|  |  | **Mean** | **Median** | **Min** | **Max** |
| --- | --- | --- | --- | --- | --- |
| **Baseline patient** | Patient code | 100 | 100 | 100 | 100 |
|  | Health Insurance code | 92 | 99 | 0 | 100 |
|  | Date of birth | 100 | 100 | 100 | 100 |
|  | Sex | 100 | 100 | 100 | 100 |
|  | Birth City | 90 | 98 | 0 | 100 |
|  | Date of death *(when appropriate)* | 100 | 100 | 92 | 100 |
|  | Country of birth | 98 | 100 | 9 | 100 |
| **Baseline onset and diagnosis** | Date of onset | 100 | 100 | 100 | 100 |
|  | Date of diagnosis | 97 | 98 | 78 | 100 |
|  | Date of first visit at the center | 97 | 100 | 14 | 100 |
|  | Presence of at least 1 out of 5 possible onset symptoms | 100 | 100 | 72 | 100 |
|  | Date of secondary progression  *(when appropriate)* | 97 | 97 | 73 | 100 |
|  | Progression from onset | 100 | 100 | 87 | 100 |
|  | Confirmed diagnosis according to McDonald 2010/2017 | 82 | 95 | 1 | 100 |
|  | MS course at onset (RR or PP) | 98 | 99 | 85 | 100 |
| **Follow-up visits** | Date of visit | 100 | 100 | 100 | 100 |
|  | MS course | 90 | 94 | 0 | 100 |
|  | EDSS | 80 | 85 | 12 | 100 |
| **Follow-up relapses** | Duration in days | 30 | 18 | 0 | 97 |
|  | Date of relapse | 98 | 100 | 0 | 100 |
|  | Presence of at least 1 functional system affected by the 8 expected | 87 | 93 | 0 | 100 |
|  | Recovery after relapse | 42 | 42 | 0 | 100 |
| **Follow-up drugs** | MS treatment specific/name of the drug | 68 | 67 | 33 | 100 |
|  | Date of start of treatment | 100 | 100 | 97 | 100 |
|  | Date of end of treatment | 100 | 100 | 99 | 100 |

**Appendix 4** Accuracy and consistency of event dates

|  |  | **Mean** | **Median** | **Min** | **Max** |
| --- | --- | --- | --- | --- | --- |
| **Baseline patient** | Date of creation record | 99 | 99 | 93 | 100 |
|  | Date of birth | 100 | 100 | 100 | 100 |
|  | Date of death *(when appropriate)* | 100 | 100 | 95 | 100 |
| **Baseline onset and diagnosis** | Date of progression *(when appropriate)* | 100 | 100 | 99 | 100 |
|  | Date of diagnosis | 97 | 98 | 78 | 100 |
|  | Date of onset | 100 | 100 | 99 | 100 |
|  | Date of first MS course | 100 | 100 | 99 | 100 |
| **Follow-up visits** | Date of first visit at the center | 96 | 99 | 14 | 100 |
|  | Date of last visit at the center | 97 | 99 | 14 | 100 |
| **Follow-up relapses** | Date of first relapse | 98 | 99 | 85 | 100 |
|  | Date of last relapse | 100 | 100 | 96 | 100 |
| **Follow-up drugs (only specific drugs)** | Date of start of treatment | 100 | 100 | 97 | 100 |
|  | Date of end of treatment | 100 | 100 | 99 | 100 |
| **Follow-up**  **MRI** | Date of first MRI | 97 | 97 | 91 | 100 |
|  | Date of last MRI | 100 | 100 | 95 | 100 |
